# BET Protein Inhibition Relieves MDSC-Mediated Immune Suppression in Chronic Lymphocytic Leukemia

**DOI:** 10.3390/hemato6020014

**Published:** 2025-05-24

**Authors:** Erin M. Drengler, Audrey L. Smith, Sydney A. Skupa, Elizabeth Schmitz, Eslam Mohamed, Dalia El-Gamal

**Affiliations:** 1 Eppley Institute for Research in Cancer and Allied Diseases, University of Nebraska Medical Center, Omaha, NE 68198, USA;; 2 College of Medicine and College of Graduate Studies, California Northstate University, Elk Grove, CA 95757, USA;; 3 Division of Hematology/Oncology, Department of Internal Medicine, College of Medicine, University of Cincinnati, Cincinnati, OH 45267, USA

**Keywords:** chronic lymphocytic leukemia (CLL), myeloid-derived suppressor cell (MDSC), BET protein inhibition, BRD4, OPN-51107 (OPN5)

## Abstract

**Background:**

Myeloid-derived suppressor cells (MDSCs) contribute to immune suppression observed in chronic lymphocytic leukemia (CLL). MDSCs are immature myeloid cells that are hijacked during development and further reprogrammed by the tumor microenvironment (TME) to harbor immune-suppressive properties and inhibit T-cell functions. Bromodomain and extraterminal domain (BET) proteins, including BRD4, are epigenetic modulators that regulate genes implicated in CLL pathogenesis and TME interactions. Previously, we investigated how the novel BET inhibitor OPN-51107 (OPN5) prevents CLL disease expansion, modulates T-cell immune function, and alters gene expression related to MDSCs. In turn, we hypothesize that BET proteins such as BRD4 regulate MDSC functions, and subsequent pharmacological inhibition of BRD4 will alleviate MDSC-mediated immune suppression in CLL.

**Methods:**

Utilizing the Eμ-TCL1 mouse model of CLL, we evaluated BRD4 protein expression in MDSCs derived from the bone marrow of transgenic and age-matched wild-type (WT) mice. We then investigated the ex vivo functionality of OPN5-treated MDSCs, expanded from Eμ-TCL1 and WT bone marrow in MDSC-supportive medium. Finally, we conducted an in vivo study utilizing the Eμ-TCL1 adoptive transfer mouse model to determine the in vivo effects of OPN5 on MDSCs and other immune populations.

**Results:**

Through the course of this study, we found that MDSCs isolated from Eμ-TCL1 mice upregulate BRD4 expression and are more immune-suppressive than their WT counterparts. Furthermore, we demonstrated ex vivo OPN5 treatment reverses the immune-suppressive capacity of MDSCs isolated from leukemic mice, evident via enhanced T-cell proliferation and IFNγ production. Finally, we showed in vivo OPN5 treatment slows CLL disease progression and modulates immune cell populations, including MDSCs.

**Conclusions:**

Altogether, these data support BET inhibition as a useful therapeutic approach to reverse MDSC-mediated immune suppression in CLL.

## 1. Introduction

Chronic lymphocytic leukemia (CLL) is a malignancy characterized by the clonal expansion and accumulation of mature CD5^+^ B-cells in the peripheral blood, bone marrow, spleen, and lymph nodes [1,2]. A highly immune-suppressive tumor microenvironment (TME) is a hallmark of CLL disease, and CLL cells are known to co-evolve with other immune cells via dynamic crosstalk [3,4]. Immune dysfunction is triggered by many different interactions in the CLL TME, including chronic antigen exposure, cell-to-cell contacts (e.g., PD-1/PD-L1), soluble mediators (e.g., IL-10, GM-CSF, G-CSF), and environmental conditions (e.g., hypoxia) [5]. Such conditions promote T-cell exhaustion and contribute to the generation of immune-suppressive cells that release suppressive signals, promote T-regulatory cells, and inhibit effective resident immune cells—all diminishing the ability for the host immune system to recognize and respond to CLL cells.

Myeloid-derived suppressor cells (MDSCs) are immature myeloid cells that are “pathologically activated” and play a significant role in T-cell suppression in cancer [6,7]. These cells are derived from monocytes or neutrophils and take on phenotypic similarities to their progenitor cells and thus were termed accordingly: monocytic-MDSCs (M-MDSCs) or granulocytic-MDSCs (G-MDSCs) [8]. However, these cells are functionally distinct from their progenitors, utilizing arginase-1 (ARG-1), nitric oxide, and reactive oxygen species, among other tools, to suppress effector immune cell function [7,9]. Given the highly immune-suppressive TME of CLL, it is unsurprising that prior works have demonstrated MDSCs to be significantly increased in the peripheral blood of CLL patients compared to healthy controls, such that the extent of their peripheral MDSC expansion is positively correlated with Rai staging and negatively correlated with time-to-treatment [10–13]. Several efforts have been made to target MDSCs in cancer, including blocking MDSC recruitment to sites of chronic inflammation, depletion of MDSCs, and inhibition of immune-suppressive activities [7,14–18]. However, the most successful efforts have targeted the immune-suppressive machinery of MDSCs, inhibiting their function and allowing the TME to recover and regain some anti-tumor immune function. Correspondingly, histone deacetylase inhibitors have been shown to influence MDSC immune-suppression in vitro and in vivo, suggesting a role for epigenetic modulation in addressing the immune-suppressive functions of MDSCs [19,20].

BET proteins (BRD2, BRD3, and BRD4) are epigenetic readers that recognize acetylated lysine residues on histones and recruit proteins such as positive transcription elongation factor complex (P-TEFb) and RNA Polymerase II to control gene expression [21,22]. BRD4 is significantly overexpressed in CLL B-cells as compared to normal B-cells, and high loads of BRD4 are found at super-enhancer regions of genes that have established roles in CLL pathogenesis, including immune function mediators and TME interactions [23,24]. BRD4 is a prominent resident of super-enhancer regions in healthy cells, in addition to cancer cells, where it helps define cell type and lineage specificity [25–27]. As an epigenetic reader and transcriptional regulator, BRD4 has been ascribed pivotal roles in inflammation, tumorigenesis, and immunity [28,29]. Our group has previously demonstrated that the BET inhibitor OPN-51107 (OPN5; previously known as PLX51107) alleviates CLL disease burden and reshapes the TME, including an observed reduction in circulating MDSCs in CLL models, prompting further investigation into the effects of BET inhibition on MDSC function in CLL [23,24]. In this study, we investigate the contribution of BET proteins, especially BRD4, to the immune-suppressive functions of MDSCs and assess whether targeting BET proteins with OPN5 can alleviate MDSC-mediated immune suppression in preclinical models of CLL.

## 2. Materials and Methods

### 2.1. Inhibitors/Drugs

Opna Bio LLC (San Francisco, CA, USA) provided the pan-BET inhibitor, OPN-51107 (OPN5). For ex vivo experiments, OPN5 was dissolved in dimethyl sulfoxide (DMSO) (Sigma-Aldrich; Saint Louis, MO, USA). For in vivo studies, OPN5 was dissolved in 10% N-methyl-2-pyrrolidone plus diluent [40% PEG400 (Sigma), 5% TPGS (ThermoFisher Scientific; Waltham, MA, USA), 5% Poloxamer 407 (Sigma), and 50% water] and administered via oral gavage [23,24].

### 2.2. Murine Studies

Eμ-TCL1 transgenic mice on a C57BL/6J background were provided by Dr. Carlo M. Croce (The Ohio State University) [30]. MDSCs were isolated from the bone marrow of Eμ-TCL1 mice (aged 4–16 months) and age-matched C57BL/6J wild-type (WT) mice for immunoblotting of BRD4 protein. For ex vivo cultures and functional studies, MDSCs were isolated from Eμ-TCL1 mice (approximately 8 months of age) with evident leukemia [>15% CD45+/CD19+/CD5+ peripheral blood lymphocytes (PBLs)] or age-matched WT mice. For adoptive transfer studies, WT mice (approximately 8 weeks old, Jackson Laboratory; Bar Harbor, ME, USA) were engrafted by intravenous (tail vein) injection with 7.5 × 10^6^ or 1.0 × 10^7^ spleen-derived lymphocytes from a moribund Eμ-TCL1 mouse as previously described [23,24]. Once leukemic cells were detectable in peripheral blood (≥5% CD45+/CD19+/CD5+ PBLs), recipient mice were randomized to receive either OPN5 (20 mg/kg) or vehicle equivalent (VEH) via oral gavage daily for up to five weeks.

Harvested spleens were homogenized into a single-cell suspension by filtration through a 70 μm filter and red blood cell (RBC) lysis using RBC Lysis Buffer (BioLegend; San Diego, CA, USA) prior to T-cell isolation or staining for flow cytometry analysis. Murine splenic T-cells were isolated using the EasySep^™^ Mouse T-cell Isolation Kit (StemCell Technologies; Vancouver, BC, Canada). Bone marrow, harvested from the tibia and femur, was subjected to RBC Lysis Buffer (BioLegend) and when appropriate, MDSCs were isolated using EasySep^™^ Mouse MDSC (CD11b+/Gr1+) negative isolation kit (StemCell Technologies; Vancouver, BC, Canada).

### 2.3. Immunoblot Analysis

Samples were lysed in RIPA buffer containing Sigma Protease and Phosphatase Inhibitor Cocktails and phenylmethyl sulfonyl fluoride (Sigma). The Pierce^™^ BCA Protein Assay (ThermoFisher) was used to quantify and equalize concentrations of protein in whole-cell lysates. Samples were then denatured at 95 °C for 5 min in loading dye containing sodium dodecyl sulfate (SDS) and separated using 6–8% acrylamide 1.5 mm SDS-PAGE gels. Gels were transferred onto 0.45 μm nitrocellulose membranes (BioRad Laboratories; Hercules, CA, USA) using Bio-Rad’s TransBlot Turbo Transfer System, then probed with primary antibodies and HRP-conjugated secondary antibodies (Bio-Rad, ThermoFisher, and Santa Cruz Biotechnology; Dallas, TX, USA). Blots were visualized on Bio-Rad’s ChemiDoc MP imager using SignalFire^™^ Elite ECL Reagent (Cell Signaling Technology; Danvers, MA, USA), WesternBright^™^ ECL (Advansta Inc.; San Jose, CA, USA), or WesternBright^™^ Sirius (Advansta) according to manufacturer’s instructions. ImageJ Fiji 1.54p software was used for densitometric band quantification [31]. Antibodies are listed in Supplemental Methods Table S1.

### 2.4. MDSC Expansion

Following bone marrow isolation, cells were resuspended in complete RPMI-1640 medium (cRPMI-1640) containing 1% L-Glutamine, 10% heat-inactivated fetal bovine serum (hi-FBS), 100 U/mL penicillin and 100 mg/mL streptomycin (P/S; Sigma), 10 mM HEPES (Sigma), 0.055 mM 2-mercaptoethanol (ThermoFisher), 0.1 mM NEAA (ThermoFisher), and 1 mM sodium pyruvate (ThermoFisher). Cells were seeded at 5.0 × 10^5^ cells/mL in a T-182 flask (ThermoFisher), and cultures were supplemented with 20 ng/mL G-CSF (cat #78014; StemCell Technologies) and 20 ng/mL GM-CSF (cat #78017; StemCell Technologies). BET inhibitor-treated conditions were exposed to 0.1 μM OPN5 at time of seeding (96 h of prolonged treatment) or 24 h before collection (short-term treatment) and compared to control conditions without the inhibitor (vehicle). All bone marrow expansions were incubated at 37 °C for four days (96 h), after which, bone marrow-derived cells were collected from the flask, washed with excess phosphate-buffered saline (PBS), and counted (~1.0 × 10^6^ cells) for flow cytometry analysis of post-expansion myeloid populations. MDSCs were isolated from the remaining cells using the EasySep^™^ Mouse MDSC (CD11b+/Gr1+) negative isolation kit (StemCell Technologies) and used for T-cell co-culture studies.

### 2.5. T-Cell:MDSC Co-Culture

For co-culture of MDSCs following 96 h ex vivo expansion of bone marrow cells, MDSCs were isolated as described above. For co-culture of MDSCs following in vivo treatment, MDSCs were isolated directly from bone marrow after sacrifice of study mice, using the EasySep^™^ Mouse MDSC (CD11b+/Gr1+) negative isolation kit (StemCell Technologies).

Healthy T-cells were isolated from splenocytes of WT mice (approximately 3–4 months of age) using EasySep^™^ Mouse T-cell Isolation Kit (StemCell Technologies). T-cells were labeled with 2 μM Cell Trace^™^ Violet (ThermoFisher) and stimulated with 10 μg/mL plate-bound anti-CD3 (clone 145-2C11, BioLegend), 1 μg/mL anti-CD28 (clone 37.51, BioLegend), and 50 ng/mL Mouse Recombinant IL-2 (StemCell Technologies). Co-cultures were seeded at 1:1, 1:1/2, 1:1/4, or 1:1/8 T-cell:MDSC ratios and incubated for three days (72 h) at 37 °C.

Following three-day co-cultures, CTV-stained T-cells were analyzed via flow cytometry to measure T-cell proliferation, defined as percent dividing after the parent CTV peak. To measure IFNγ production, cultures were stimulated with 1X PMA/Ionomycin (BioLegend) for a total of 6 h, with 1X Brefeldin A (BioLegend) added for the final 5 h. Cells were then collected and incubated with fluorochrome-labeled antibodies against extracellular markers or intracellular IFNγ analyzed for flow cytometry analysis.

### 2.6. Flow Cytometry

A list of fluorochrome-labeled antibodies and gating strategies are provided in Supplemental Methods Table S2, and Supplemental Methods Figures S1 and S2.

Murine spleen, bone marrow, or ex vivo cultured cells (~1–2 × 10^6^) were collected, washed with PBS, re-suspended in PBS/2% hi-FBS, and incubated with fluorochrome-labeled antibodies (100 μL total volume) at 4 °C for 30 min. When appropriate, cells were fixed with Fixation Buffer, or Cyto-Fast^™^ Fix/Perm Buffer Set, for intracellular staining per manufacturer’s protocol (BioLegend). Data acquisition was performed on an LSRII (BD Biosciences; Franklin Lakes, NJ, USA), LSRFortessa X-50 (BD Biosciences), or NovoCyte 2060R (Agilent; Santa Clara, CA, USA) cytometer. Data were analyzed using NovoExpress v1.3.0 (Agilent) or Kaluza v2.1 (Beckman Coulter; Brea, CA, USA). Singlet lymphocytes were gated by forward and side scatter, and live cells were gated by Live/Dead Near IR (ThermoFisher) negative staining. We ensured correct compensation, proper acquisition setup, and set positive gates based on appropriate fluorescence minus one (FMO) controls. Results are expressed as the proportion of cells expressing antigens of interest (percent gated positive). CTV gating is expressed as percent dividing to the left of the parent CTV peak.

To monitor leukemic disease burden in mice, approximately 25 μL blood was obtained from the submandibular vein. Whole blood was incubated with fluorochrome-labeled antibodies (in a maximum volume of 20 μL) at 4 °C for 20 min, then lysed using RBC Lysis Buffer (BioLegend) per manufacturer’s protocol prior to flow cytometry analysis.

### 2.7. Cytotoxicity Assay

CD8+ T-cells were isolated from spleens of treated adoptive transfer mice using the EasySep^™^ Mouse CD8+ T Cell Isolation Kit (StemCell Technologies). CLL B-cells (target) were isolated from pooled splenocytes of VEH-treated mice using the EasySep^™^ Mouse Pan-B Cell Isolation Kit. Isolated B-cells were labeled with 2 μM CTV (ThermoFisher), then co-cultured with CD8+ T-cells in a 96-well plate at a 1:1 effector-to-target ratio (2 × 10^5^ of each cell type) in the presence of 10 μg/mL anti-CD3 (clone 145-2C11, BioLegend), 5 ng/mL Mouse Recombinant IL-15 (StemCell Technologies), and 50 ng/mL Mouse Recombinant IL-2 (StemCell Technologies) for 16 h at 37 °C. Cells were then collected and labeled with Zombie NIR Fixable Viability dye (BioLegend) and analyzed via flow cytometry for the number of dead B-cells (CTV+/Zombie NIR+) per 100 viable CD8+ T-cells (CTV−/Zombie NIR−) as previously described [24].

### 2.8. Statistics

Data are reported as mean ± standard error of the mean (SEM). Statistically significant differences between two groups were determined using an unpaired Mann–Whitney U test or paired Wilcoxon matched-pairs signed rank test. Statistically significant differences among more than two groups were determined using Kruskal–Wallis one-way ANOVA tests coupled with Dunn’s post hoc analysis. All statistical analyses were performed with GraphPad Prism v10.4.2 (Boston, MA, USA). *p* values < 0.05 were considered significant.

## 3. Results

### 3.1. CLL-Associated MDSCs Overexpress BRD4

**Figure 1.**
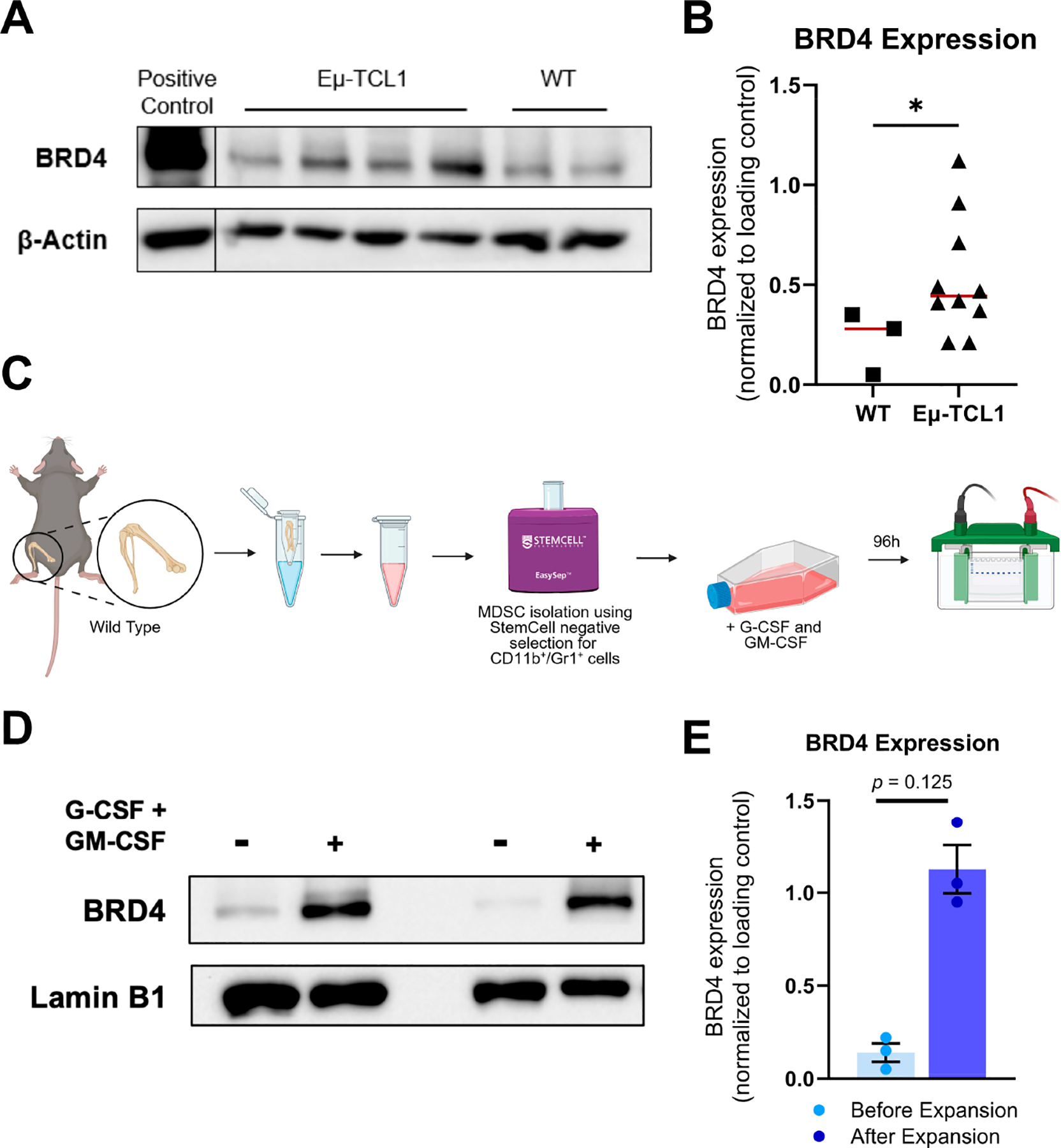
BRD4 is overexpressed in MDSCs derived from Eμ-TCL1 mice and expanded MDSCs. (**A**) MDSCs were isolated from C57BL/6J wild-type (WT) or Eμ-TCL1 mice (4–16 months of age), and whole-cell lysates were directly analyzed for expression of BRD4 via immunoblot. β-Actin was used as a loading control. A representative immunoblot is shown. (**B**) Densitometric quantification of BRD4 from immunoblots of MDSCs isolated from WT (n = 3) or Eμ-TCL1 mice (n = 10). Expression relative to loading control. Red line represents mean. (**C**) Workflow schematic for isolating CD11b+/Gr1+ MDSCs from WT murine bone marrow using StemCell EasySep^™^ Mouse MDSC (CD11b+/Gr1+) negative isolation kit and expanding in MDSC-supportive medium containing 20 ng/mL G-CSF and 20 ng/mL GM-CSF for 96 h for immunoblot analysis. (**D**) A representative immunoblot of WT CD11b+/Gr1+ cells before and after expansion with MDSC-supportive medium is shown. Whole-cell lysates were collected and analyzed for expression of BRD4 via immunoblot. Lamin B1 was used as a loading control. (**E**) Densitometric quantification of MDSC BRD4 protein levels relative to loading control (n = 3 experimental replicates). Data are presented as mean ± SEM. Unpaired Mann–Whitney U tests were used to determine the significance between WT and Eμ-TCL1 BRD4 expression (**A**,**B**). Paired Wilcoxon tests were used to determine the significance between BRD4 expression in CD11b+/Gr1+ MDSCs before and after expansion (**D**,**E**); * *p* < 0.05. WT = wild-type, G-CSF = granulocyte-colony stimulating factor, GM-CSF = granulocyte/macrophage-colony stimulating factor.

The Eμ-TCL1 mouse model is a well-established transgenic model that recapitulates human CLL disease course, TME interactions, immune dysfunction, and response to therapeutics [32–35]. We first set out to establish the expression of BRD4 in MDSCs using immunoblot analysis, for which MDSCs (CD11b+/Gr1+ cells) were isolated from the bone marrow of Eμ-TCL1 mice and control WT mice. Higher levels of BRD4 protein were observed in MDSCs isolated from Eμ-TCL1 mice, compared to age-matched WT mice (Figure 1A,B). Furthermore, when MDSCs isolated from WT mice were expanded in MDSC-supportive medium (cRPMI-1640 containing G-CSF and GM-CSF) (Figure 1C), they demonstrated higher BRD4 levels in comparison to MDSCs isolated from the same mouse, prior to expansion with growth factors (Figure 1D,E), implying a potential role for BRD4 in the induction or maintenance of MDSCs.

### 3.2. BET Protein Inhibition Alters MDSC Differentiation and Reduces Their Immune-Suppressive Activity Ex Vivo

While CD11b and Gr1 are commonly accepted as markers for murine MDSCs, MDSC research lacks unique markers to definitively identify “bona fide” MDSCs. Therefore, MDSC research is dominated by functional testing of MDSC immune-suppressive activity to define “bona fide” MDSCs. Thus, we utilized T-cell immune suppression assays to evaluate MDSC functionality by their ability to suppress healthy T-cell proliferation or IFNγ production.

To investigate the effect of BET inhibition on the generation of MDSCs, whole bone marrow aspirates (after RBC lysis) were expanded in MDSC-supportive medium to which OPN5 (0.1 μM) was added either at the beginning of expansion for the entire 96 h (prolonged OPN5 treatment), or in the final 24 h of expansion (short-term OPN5 treatment), and compared to normal expansion conditions (no OPN5/vehicle control) (Supplemental Data Figure S1A). Cells were then evaluated by flow cytometry for expression of MDSC phenotypic surface markers (Supplemental Methods Figure S2). With the addition of OPN5 treatment, the percentage of M-MDSCs was reduced in expansion cultures of bone marrow cells derived from both WT and Eμ-TCL1 mice, within 24 h (Supplemental Data Figure S1B). When expanded cultures received prolonged OPN5 treatment, the percentage of M-MDSCs in both WT- and Eμ-TCL1-derived expansions further decreased compared to the vehicle control (*p* < 0.001) (Supplemental Data Figure S1B). However, compared to the vehicle control, the percentage of G-MDSCs was significantly increased in Eμ-TCL1-derived expansions (Supplemental Data Figure S1C). OPN5 treatment appears to impair the expansion of M-MDSCs, thus shifting the balance of G-MDSCs and M-MDSCs within the ex vivo culture model. Importantly, patients with CLL demonstrate higher percentages of M-MDSCs than G-MDSCs in the peripheral blood, and M-MDSCs tend to be more immune-suppressive than their G-MDSC counterparts [10–12,32].

**Figure 2.**
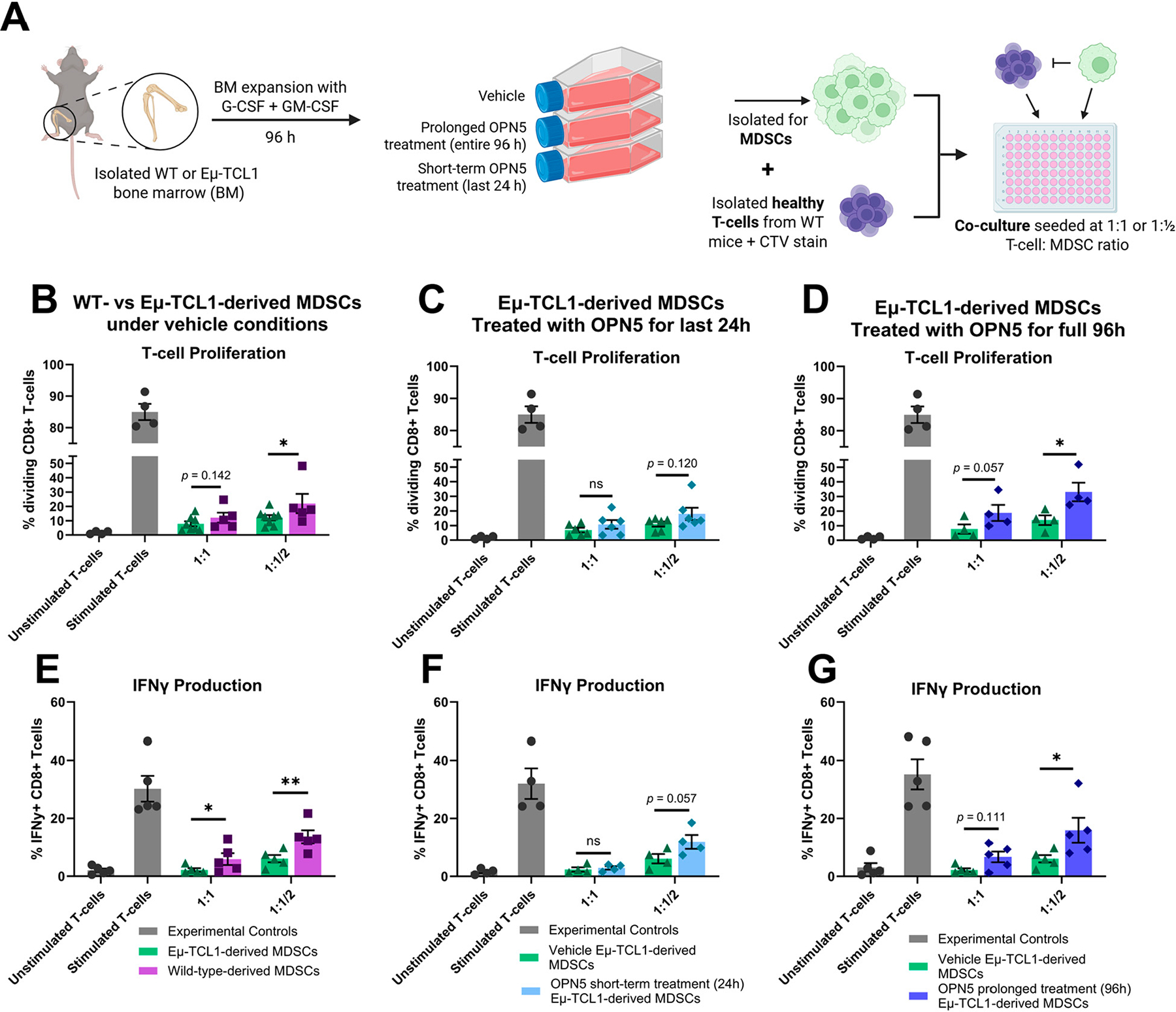
Ex vivo treatment with OPN5 alleviates MDSC-mediated suppression of T-cell function. (**A**) Workflow schematic demonstrating ex vivo expansion conditions and treatment with OPN5 followed by co-culture with healthy T-cells isolated from C57BL/6J wild-type (WT) mice. All conditions received MDSC-supportive cRPMI-1640 medium containing 20 ng/mL G-CSF and 20 ng/mL GM-CSF. BET inhibitor-treated conditions included prolonged OPN5 treatment, which received 0.1 μM OPN5 at the beginning of the expansion (96 h total), and short-term OPN5 treatment, which received 0.1 μM OPN5 for the final 24 h of expansion. Comparisons were made to bone marrow expanded in the absence of OPN5 (vehicle). Following expansion, OPN5 was washed off prior to T-cell functional assays. MDSCs were isolated via negative selection and co-cultured with T-cells to evaluate suppression of T-cell proliferation and IFNγ production. (**B**) Eμ-TCL1-derived bone marrow (n = 8) and WT-derived bone marrow (n = 5) expanded in MDSC-supportive medium. At end of 96 h expansion, MDSCs were isolated, and co-cultured with CTV-labeled healthy T-cells (isolated from WT mouse spleens) at 1:1 or 1:1/2 T-cell:MDSC ratio. To stimulate T-cell proliferation, 10 mg/mL plate-bound murine anti-CD3, 1 mg/mL murine anti-CD28, and 50 ng/mL murine IL-2 were added. The percentage of dividing T-cells is shown. Experimental controls (dark grey bars) include monocultures of unstimulated healthy T-cells and stimulated T-cells (anti-CD3/anti-CD28 with IL-2). (**C**) Eμ-TCL1-derived bone marrow expanded in MDSC supportive medium were subject to short-term BET inhibitor treatment (0.1 μM OPN5 for the last 24 h of expansion) and compared to vehicle control (n = 6 experimental replicates). (**D**) Eμ-TCL1-derived bone marrow expanded in MDSC supportive medium were subjected to prolonged BET inhibitor treatment (0.1 μM OPN5 for the full 96 h of expansion) and compared to vehicle control (n = 4 experimental replicates). (**E**) Percentage of IFNγ + CD8+ T-cells following co-culture with WT or Eμ-TCL1-derived MDSCs expanded in MDSC-supportive medium (n = 5 experimental replicates). (**F**) Percentage of IFNγ + CD8+ T-cells following co-culture with Eμ-TCL1-derived MDSCs expanded in MDSC-supportive medium under vehicle control conditions or expanded in the presence of OPN5 (0.1 μM) for 24 h (n = 4 experimental replicates). (**G**) Percentage of IFNγ + CD8+ T-cells following co-culture with Eμ-TCL1-derived MDSCs expanded in MDSC-supportive medium under vehicle control conditions or expanded in the presence of OPN5 (0.1 μM) for 96 h (n = 5 experimental replicates). Data are presented as mean ± SEM. Unpaired Mann–Whitney U tests were used to determine the significance between treatment groups; ns = not significant, * *p* < 0.05, ** *p* < 0.01. WT = wild-type, G-CSF = granulocyte-colony stimulating factor, GM-CSF = granulocyte/macrophage-colony stimulating factor, OPN5 = OPN-51107, CTV = cell trace violet.

Following the expansion of bone marrow-derived cells with or without OPN5 treatment, MDSCs were isolated for subsequent co-cultures along with healthy anti-CD3/anti-CD28 stimulated T-cells to test their immune-suppressive capacity (Figure 2A). OPN5 was washed out of treated cultures before the set-up of T-cell suppression assays, to avoid observing direct effects of OPN5 on T-cell function. First, MDSCs derived from expansion cultures of WT or Eμ-TCL1 bone marrow without OPN5 treatment (vehicle) were compared (Figure 2B,E). Eμ-TCL1-derived MDSCs were more suppressive than WT-derived MDSCs, as indicated by stronger impairment of T-cell proliferation (Figure 2B). Eμ-TCL1-derived MDSC co-cultures demonstrated decreased CD8+ T-cell division at 1:1 (approaching statistical significance, *p* = 0.142) and 1:1/2 (*p* < 0.05) T-cell:MDSC ratios as compared to WT-derived MDSCs under vehicle control conditions (Figure 2B).

Utilizing the same experimental set-up (Figure 2A), Eμ-TCL1-derived MDSCs under vehicle control conditions were compared to 96 h- or 24 h-OPN5-treated Eμ-TCL1-derived MDSCs. MDSC immune-suppressive function begins to decrease by 24 h of OPN5 treatment (Figure 2C). Compared to vehicle control conditions, OPN5-treated MDSC co-cultures seeded at 1:1/2 ratio demonstrated increased CD8+ T-cell proliferation (approaching significance, *p* = 0.12) (Figure 2C). When Eμ-TCL1 bone marrow expansion was treated with OPN5 for the full 96 h, MDSC immune-suppressive activity was significantly reduced (Figure 2D). Even at higher MDSC proportions (1:1 T-cell:MDSC ratio), Eμ-TCL1-derived MDSCs subjected to prolonged OPN5 treatment had a diminished effect on dividing CD8+ T-cells, as compared to their vehicle counterparts (*p* = 0.057) (Figure 2D). Similarly, at the 1:1/2 T-cell:MDSC ratio, Eμ-TCL1-derived MDSCs treated with OPN5 for 96 h suppressed CD8+ T-cell division significantly less than their vehicle counterparts (Figure 2D).

To further validate the reduced MDSC immune-suppressive activity witnessed with BET inhibitor treatment, in conjunction with T-cell proliferation, co-cultured T-cell IFNγ production was evaluated. Decreased IFNγ production by T-cells is indicative of poor effector function and immune suppression. Similar trends in immune-suppressive activity to that measured by T-cell proliferation were witnessed for IFNγ production under all conditions (Figure 2E–G). Eμ-TCL1-derived MDSCs significantly suppressed T-cell IFNγ production to a greater extent than WT-derived MDSCs under vehicle control conditions when co-cultured at 1:1 and 1:1/2 T-cell:MDSC ratios (Figure 2E). Additionally, OPN5 treatment begins to alleviate the immune-suppressive burden of Eμ-TCL1-derived MDSCs within 24 h, as demonstrated by increased T-cell IFNγ production (*p* = 0.057) at the 1:1/2 T-cell:MDSC ratio compared to vehicle control (Figure 2F). Finally, Eμ-TCL1-derived MDSC immune suppression was reduced with prolonged BET inhibition (full 96 h OPN5 treatment) at 1:1 (approaching statistical significance, *p* = 0.111) and 1:1/2 (*p* < 0.05) T-cell:MDSC ratio when compared to vehicle control (Figure 2G).

To determine if this effect was disease-specific, we also evaluated WT-derived BM expansions with short-term (24 h) or prolonged (96 h) OPN5 treatment in co-culture with healthy CTV-stained T-cells. Short-term treatment with OPN5 did not result in alleviation of T-cell suppression, and prolonged OPN5 treatment alleviated T-cell suppression only at the lower T-cell:MDSC ratio (1:1/2) (Supplementary Data Figure S2A,B).

Overall, these data demonstrate that BRD4 inhibition by OPN5 clearly alleviates the immune-suppressive capacity of Eμ-TCL1-derived MDSCs, indicating a role for BRD4 and other BET proteins in MDSC-mediated immune suppression in CLL.

### 3.3. BET Protein Inhibition Reduces MDSC Immune-Suppressive Activity In Vivo

**Figure 3.**
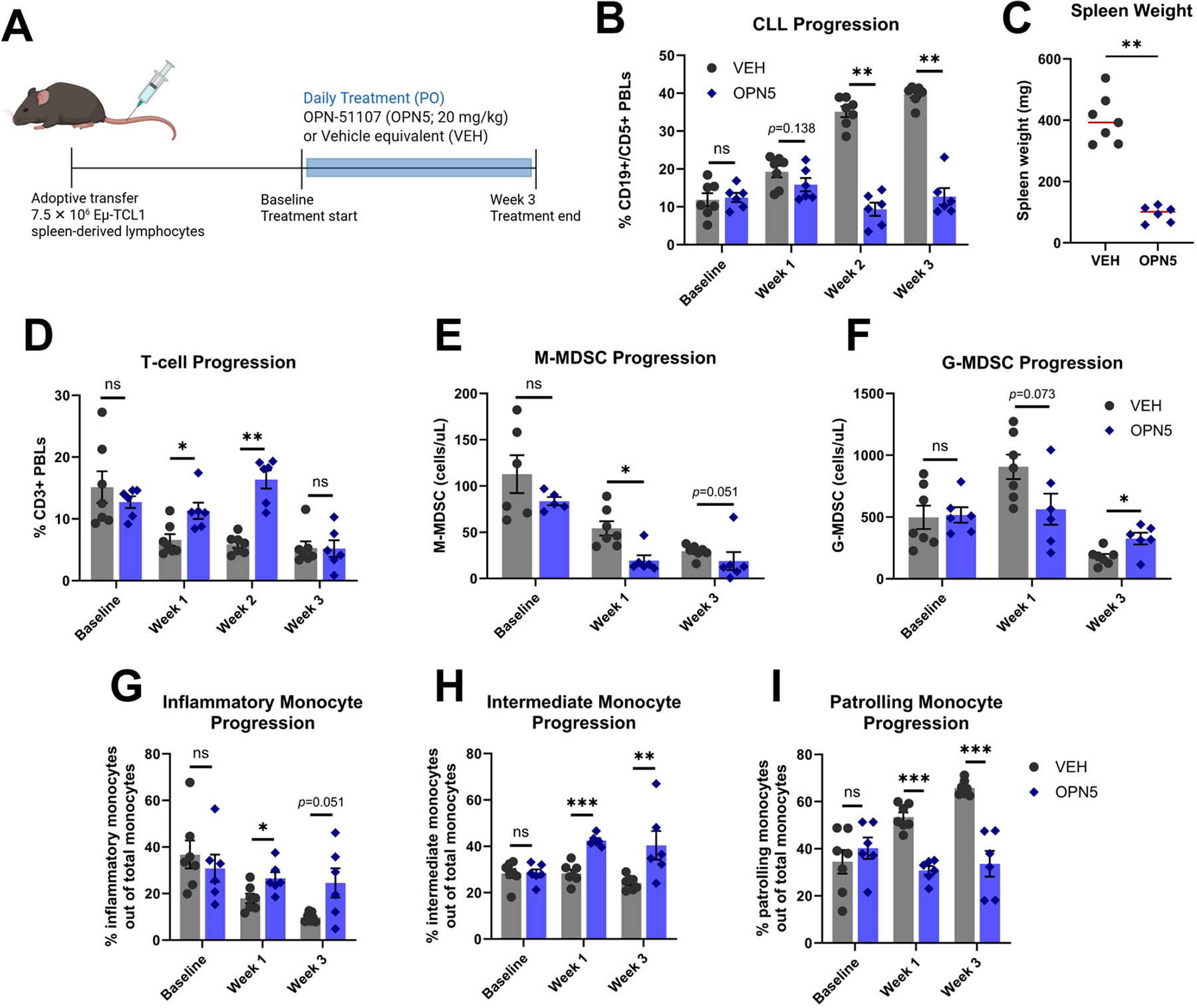
In vivo OPN5 treatment influences CLL progression and microenvironment. (**A**) Schematic of Eμ-TCL1 AT mouse study. Approximately three weeks following CLL engraftment, mice were randomly assigned to treatment groups at CLL disease onset (>5% CD45+/CD19+/CD5+ PBLs). Mice received daily treatment of 20 mg/kg OPN5 (n = 6) or vehicle equivalent (n = 7) via oral gavage. (**B**) Percentage of CD45+/CD19+/CD5+ PBLs (CLL-like B-cells) are presented before treatment (baseline) and across three weeks of treatment. (**C**) Spleen weight in milligrams following study end. Red line represents mean. (**D**) Percentage of T-cells (CD45+/CD3+ cells) are presented before treatment (baseline) and across three weeks of treatment. (**E**–**I**) Flow cytometry analysis of peripheral blood myeloid subsets (MDSCs and monocytes) was conducted at baseline and weeks 1 and 3 post-treatment: (**E**) M-MDSCs gated as CD45+/CD11b+/Ly6C+/Ly6G- cells on lineage negative (CD19−/CD3−) cells. (**F**) G-MDSCs gated as CD45+/CD11b+/Ly6Clo/Ly6G+ cells on lineage negative (CD19−/CD3−) cells. (**G**–**I**) Subsets of monocytes are gated as CD19−/CD3−/CD11b+/Ly6G-then subdivided by Ly6C and CD43 expression: inflammatory (Ly6Chi/CD43lo), intermediate (Ly6Cmed/CD43med), or patrolling (Ly6Clo/CD43hi). Data are presented as mean ± SEM. Unpaired Mann–Whitney U tests were used to determine significant differences between VEH and OPN5 groups; ns = not significant, * *p* < 0.05, ** *p* < 0.01, *** *p* < 0.001. OPN5 = OPN-51107, VEH = vehicle equivalent, PBLs = peripheral blood lymphocytes.

Expanding on our ex vivo findings, we evaluated the effect of in vivo OPN5 treatment on the immune-suppressive activity of CLL-associated MDSCs. The aggressive Eμ-TCL1 adoptive transfer (AT) model was employed by engrafting 7.5 × 10^6^ spleen-derived lymphocytes from a moribund Eμ-TCL1 mouse into recipient WT mice by intravenous (tail vein) injection. Mice were bled weekly to assess CLL development via flow cytometry analysis of PBLs. Upon CLL disease onset (>5% CD45+/CD19+/CD5+ PBLs), mice were randomly assigned to receive OPN5 or vehicle equivalent (VEH) treatment daily for three weeks (Figure 3A). At study end, treatment groups displayed disparate disease levels (VEH: 39.77 ± 0.91% vs. OPN5: 12.74 ± 2.21% CD45+/CD19+/CD5+ PBLs) (Figure 3B). This difference in disease level was reflected in the spleen weight of mice from each treatment group such that untreated disease in the VEH-treated mice resulted in CLL-like marked splenomegaly that is significantly reduced with OPN5 treatment (Figure 3C). The percentage of T-cells in the peripheral blood of OPN5-treated mice remained significantly higher than that of VEH-treated mice for most of the treatment course (Figure 3D). Absolute counts of MDSCs were measured in the peripheral blood bi-weekly. OPN5 treatment reduced absolute counts of M-MDSCs in the peripheral blood, and by the end of treatment, absolute counts of G-MDSCs were higher in the peripheral blood of OPN5-treated mice as compared to VEH-treated mice (Figure 3E,F). Extending our investigation to other subsets of myeloid cells in the leukemic microenvironment, the progression of inflammatory, intermediate, and patrolling monocytes in the peripheral blood were evaluated. Inflammatory and intermediate monocytes increased over time with OPN5 treatment (Figure 3G,H), while patrolling monocyte levels decreased over time with OPN5 treatment (Figure 3I) [24,36].

We confirmed these results in another Eμ-TCL1 AT study (Supplementary Data Figure S3) wherein 1 × 10^7^ spleen-derived lymphocytes from a serial-engrafted leukemic donor mouse were engrafted in each recipient WT mouse, cultivating more aggressive disease progression. These mice were treated with VEH or OPN5 (20 mg/kg, PO) for 5 weeks. At the end of treatment, mice that received OPN5 displayed a significantly lower percentage of CD45+/CD19+/CD5+ PBLs than mice that received VEH (Supplementary Data Figure S3A). M-MDSCs were also markedly reduced in the peripheral blood of OPN5-treated mice, while G-MDSC populations remained unchanged (Supplementary Data Figure S3B).

**Figure 4.**
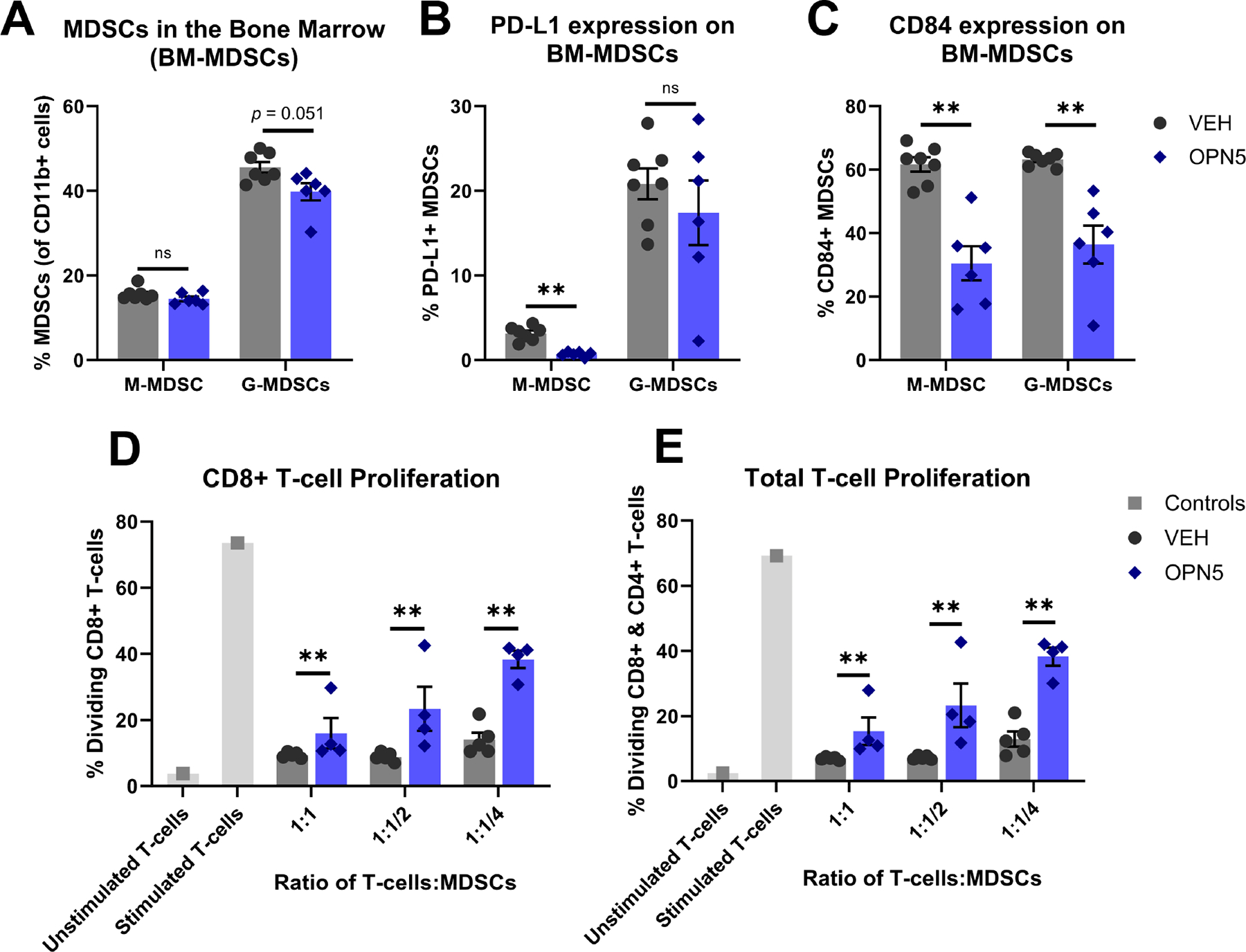
In vivo OPN5 treatment modulates MDSCs in the bone marrow physically and functionally. (**A**) Percentage of M-MDSCs in the bone marrow gated as CD45+/CD19−/CD3−/CD11b+/Ly6C+/Ly6G−. Percentage of G-MDSCs in the bone marrow gated as CD45+/CD19−/CD3−/CD11b+/Ly6Clo/Ly6G+. (**B**) Percentage of PD-L1+ bone marrow M-MDSCs and G-MDSCs. (**C**) Percentage of CD84+ bone marrow M-MDSCs and G-MDSCs. (**D**,**E**) MDSCs (CD11b+/Gr1+) isolated from the bone marrow of VEH- (n = 5) and OPN5- (n = 4) treated mice directly after sacrifice were co-cultured with healthy T-cells to determine MDSC suppressive function. Healthy T-cells were isolated from a pool of splenocytes from 3-month-old WT mice and labeled with CTV for T-cell functionality assays. Co-cultures were seeded at 1:1, 1:1/2, and 1:1/4 T-cell:MDSC ratios, and stimulated with 10 mg/mL plate-bound murine anti-CD3, 1 mg/mL murine anti-CD28, and 50 ng/mL murine IL-2. Experimental controls (light grey bars) include monocultures of unstimulated healthy T-cells and T-cells stimulated with anti-CD3/anti-CD28 in the presence of IL-2. The percentage of CD8+ or total (CD4+ and CD8+) T-cells that underwent cell division is shown. Data are presented as mean ± SEM. Unpaired Mann–Whitney U tests were used to determine significant differences between VEH-treated (n = 5–7) and OPN5-treated (n = 4–6) groups; ns = not significant, ** *p* < 0.01. BM-MDSCs = bone marrow MDSCs, VEH = vehicle equivalent, OPN5 = OPN-51107.

To further assess the effect of OPN5 treatment on MDSCs in vivo, we turned to an important TME niche in CLL: the bone marrow. At end of study, bone marrow cells from treated mice were evaluated for MDSC burden via flow cytometry. Overall, M-MDSC and G-MDSC burden in the bone marrow was not significantly changed between treatment groups (Figure 4A). Despite minimal changes in the percentage of MDSCs in the bone marrow niche, the MDSCs present in the bone marrow of OPN5-treated mice displayed markedly decreased expression of immune-inhibitory molecules commonly found on MDSCs (PD-L1 and CD84). M-MDSCs demonstrated a significant decrease in PD-L1 expression with OPN5 treatment (*p* < 0.01), and both M-MDSC and G-MDSC populations from OPN5-treated mice had significantly less CD84 expression compared to those from VEH-treated mice (*p* < 0.01) (Figure 4B,C). To determine if in vivo BET inhibitor treatment impacted MDSC functionality, MDSCs were isolated directly from the bone marrow of treated mice and immediately co-cultured with CTV-stained healthy anti-CD3/anti-CD28 stimulated T-cells—derived from spleens of WT mice—to determine immune-suppressive activity via inhibition of T-cell proliferation. MDSCs from OPN5-treated mice were less capable of suppressing CD8+ and total T-cell proliferation at all co-culture ratios (1:1, 1:1/2, 1:1/4 T-cell:MDSC ratios) evaluated (Figure 4D,E). These data, in conjunction with decreased expression of immune-suppressive markers, PD-L1 and CD84, indicate in vivo OPN5 treatment functionally reverses the immune-suppressive activity and mechanisms of CLL-associated MDSCs.

Again, concordant results were observed in a separate AT study utilizing a more aggressive donor (Supplementary Data Figure S3). In this study, the abundance of M-MDSCs in the bone marrow decreased with OPN5 treatment, and OPN5-treated mice demonstrated a slight decrease in G-MDSCs compared to VEH-treated mice (Supplementary Data Figure S3C). Even though MDSCs from these mice were capable of suppressing T-cell proliferation at low T-cell:MDSC ratios (1:1/4 and 1:1/8), OPN5 treatment significantly alleviated their suppressive capacity (Supplementary Data Figure S3D).

**Figure 5.**
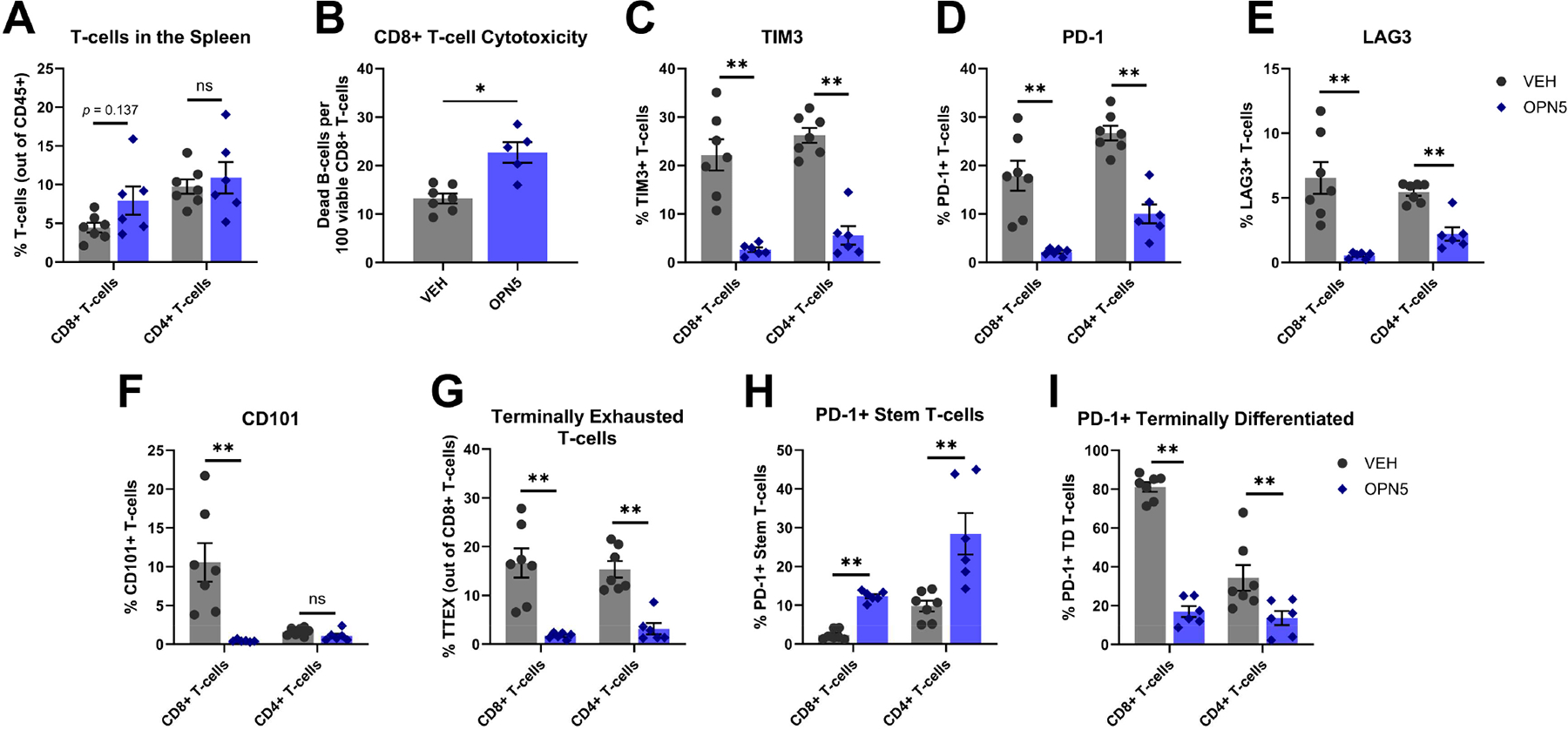
In vivo OPN5 treatment modulates T-cell functions. (**A**) Abundance of T-cell subsets found in the spleen of mice treated for 3 weeks with OPN5 (n = 6) or VEH (n = 7). T-cells gated as CD45+/CD19−/CD8+ or CD4+. (**B**) Cytotoxic capacity of splenic CD8+ T-cells co-cultured with CFSE-labeled splenic CLL B-cells (pooled from VEH-treated leukemic mice) at a 2:1 effector-to-target ratio. The number of dead CLL B-cells per 100 viable CD8+ T-cells following 18 h co-culture is shown. (**C**–**E**) Percentages of T-cells expressing immune inhibitory receptors/checkpoint molecules (TIM3, PD-1, LAG3). (**F**) Percentage of T-cells expressing exhaustion marker CD101. (**G**) Percentage of terminally exhausted CD8+ T-cells (T_TEX_) gated as PD-1hi/TIM3hi. (**H**,**I**) Percentages of progenitor/stem-like (stem; PD-1+/TIM3−/Ly108+) and terminally differentiated (TD; PD-1+/TIM3+/Ly108−) CD8+ and CD4+ T-cells. Data are presented as mean ± SEM. Unpaired Mann–Whitney U tests were used to determine significant difference between VEH-treated and OPN5-treated groups; ns = not significant, * *p* < 0.05, ** *p* < 0.01. VEH = vehicle equivalent, OPN5 = OPN-51107, T_TEX_ = terminally exhausted, Stem = progenitor/stem-like, TD = terminally differentiated.

Previously, our group has demonstrated that OPN5 treatment reduced T-cell dysfunction in CLL [24]; however, we wanted to expand this characterization, especially considering the decreased T-cell suppressive activity of MDSCs in OPN5-treated mice. Overall, CD8+ and CD4+ T-cell subsets in the spleen were not significantly changed with OPN5 treatment (Figure 5A). However, similar to the MDSC subsets in the bone marrow, the T-cells present in OPN5-treated mice were functionally different from those of VEH-treated mice. Splenic CD8+ T-cells from OPN5-treated mice were more effective than those from VEH-treated mice at directly killing leukemic B-cells (Figure 5B). CD8+ and CD4+ T-cells from OPN5-treated mice also exhibited decreased expression of common immune checkpoint molecules (TIM3, PD-1, and LAG3) (Figure 5C–E) [37–39]. CD8+ T-cells from the spleens of OPN5-treated mice exhibited decreased expression of the exhaustion marker CD101 as compared to T-cells from VEH-treated mice (Figure 5F) [40,41]. Furthermore, PD-1 co-expression with other checkpoint molecules is a common pre-requisite for classifying exhausted T-cells [39]. To characterize the extent of splenic T-cell exhaustion, the co-expression of PD-1 and TIM3 on CD8+ T-cells was assessed. PD-1+/TIM3+ T-cells were defined as terminally exhausted T-cells (T_TEX_) [42,43]. As expected, OPN5-treated mice exhibited a significantly decreased population of both CD8+ and CD4+ T_TEX_ cells (Figure 5G). Exhausted T-cells were further characterized by expression of Ly108 (SLAMF6), a surrogate marker for the transcription factor TCF1 which promotes T-cell progenitor function [42–44]. PD1+ CD8+ or CD4+ T-cells were evaluated for their expression of TIM3 and Ly108, indicative of either “stem-like” (TIM3−/Ly108+) or “terminally differentiated” (TIM3+/Ly108−) phenotypes [42,43]. OPN5-treated mice exhibited a significantly increased percentage of “stem-like” T-cells coupled with a markedly decreased percentage of “terminally differentiated” T-cells compared to VEH-treated mice (Figure 5H,I). These data suggest potential for re-invigoration of CLL-associated T-cells with OPN5 treatment. Overall, these data demonstrate OPN5 treatment lessens T-cell immune dysfunction in the splenic microenvironment, reinforcing that BET protein inhibition repairs the suppressive microenvironment in CLL.

## 4. Discussion

In this study, we demonstrated that BRD4 is upregulated in CLL-associated MDSCs and pharmacological inhibition with the novel BET inhibitor, OPN5, alleviated MDSC-mediated immune-suppression in ex vivo and in vivo settings. To begin, we found that MDSCs isolated from Eμ-TCL1 mice exhibited increased BRD4 expression and immune-suppressive capacities (measured via T-cell proliferation and IFNγ production assays) as compared to MDSCs from age-matched WT mice. Interestingly, bone marrow-derived MDSCs from healthy mice demonstrated increased expression of BRD4 protein when expanded in the presence of MDSC-supportive medium (cRPMI-1640 supplemented with G-CSF and GM-CSF), suggesting a potential role for BRD4 in the development or maintenance of MDSCs and warranting further investigation into its role in MDSC biology and differentiation. Along with its importance in the development and maintenance of cell phenotypes, BRD4 regulates various oncogenic proteins such as MYC, and modulates critical cell signaling pathways like the JAK/STAT and NF-κB pathways, all of which are important in the induction of MDSCs [7,45]. Therefore, MDSCs may utilize BRD4 to govern and/or sustain transcriptional programs underlying their immune-suppressive behavior. The potential role and regulation of BRD4 in MDSCs holds translational relevance and prompts further study.

In CLL, immune suppression contributes to negative outcomes in patients, as ineffective immune responses directly contribute to reduced disease clearance and secondary complications of immune dysfunction, such as susceptibility to infection [1,2]. M-MDSCs are widely found to be over-abundant in the peripheral blood of patients with CLL and are more immune-suppressive than G-MDSCs, due to M-MDSC’s prolonged immune impacts (e.g., soluble factors) as compared to G-MDSC’s shorter-lived impacts (e.g., ROS production) [10–12,32]. Our results indicate that, in both ex vivo and in vivo contexts, BET inhibition influences the balance of MDSC populations. In murine bone marrow cell cultures treated ex vivo with OPN5, M-MDSC populations decreased with as little as 24 h of treatment and continued to trend downward with prolonged OPN5 treatment (96 h). Simultaneously, G-MDSC percentages increased with 96 h of ex vivo OPN5 treatment. This outcome is mirrored in the peripheral blood of AT Eμ-TCL1 mice, where M-MDSC percentages decreased and G-MDSC percentages increased over time with OPN5 treatment as compared to VEH-treated counterparts. This could be attributed to the lower transcriptional activity of G-MDSCs [46], which could lessen the impact of transcriptional inhibitors like OPN5 on this subset. Although both MDSC populations would ideally decrease with effective immunomodulatory anti-cancer therapy, it is encouraging that OPN5 directly impacts M-MDSCs—an aggressively immune-suppressive MDSC population linked to negative outcomes in patients with CLL [10–12,32].

To test how OPN5 treatment directly modulates MDSC function, we excluded secondary effects of OPN5′s anti-cancer activity by first treating murine-derived MDSCs in ex vivo cultures. In this experimental setting, we pre-conditioned bone marrow cell cultures with BET inhibitor treatments (short-term vs. prolonged OPN5 exposure). Thereafter, OPN5 was washed off, and these conditioned—-or reprogramed MDSCs—-were used in T-cell immune suppression assays. Exposure to OPN5 for as little as 24 h and up to 96 h reversed the capacity of MDSCs to suppress T-cell proliferation and IFNγ production, demonstrating that BET inhibition has a direct impact on MDSC function, rendering MDSCs less immune-suppressive.

Once established, we sought to further investigate this phenomenon in an aggressive mouse model of CLL, namely the AT Eμ-TCL1 model. Previous studies from our group have demonstrated the anti-CLL effects of OPN5 treatment in the AT Eμ-TCL1 model [23,24]. Through targeted gene expression profiling (PanCancer iO360, Nanostring) of key CLL microenvironment niches, we discovered that OPN5 treatment of AT Eμ-TCL1 mice reduced the expression of genes related to MDSC function (e.g., *Cd36*, *Cd38*, *Cd84*, *Entpd*, and *Adora2*) [24]. In concordance with our previous studies, we demonstrated that in vivo OPN5 treatment decreased CLL-like disease progression and expansion of immune-suppressive cells in the peripheral blood. However, in this study, we further explored the effects of in vivo OPN5 treatment on the different MDSC subsets and established the therapeutic benefit of BET inhibition on re-wiring MDSCs to become less suppressive. At the conclusion of the murine study, we found MDSC populations in the bone marrow were not significantly changed in number; however, OPN5 treatment incited important phenotypic and functional changes in these MDSCs. Phenotypically, MDSCs isolated from the bone marrow of OPN5-treated mice exhibited decreased expression of PD-L1 and CD84—important immune-inhibitory molecules that contribute to the suppression capabilities of MDSCs [7,47]. The PD-1/PD-L1 axis has been extensively studied in the regulation of immune suppression in CLL, playing important roles in suppressing T-cell function in the TME [36,48–50]. CD84 expression on MDSCs has been shown to upregulate PD-L1 expression on CLL B-cells and MDSCs, further contributing to MDSC-mediated T-cell suppression in CLL [51]. Functionally, MDSCs isolated from the bone marrow of OPN5-treated mice were less capable of suppressing T-cell proliferation and demonstrate that the effects of OPN5 on MDSC activity are maintained in vivo, highlighting the therapeutic potential of BRD4 targeting in re-programming MDSCs to enhance immune-mediated tumor control in CLL.

OPN5 has been used in clinical trials as a monotherapy in Phase I/II trials (NCT04910152, NCT02683395; clinicaltrials.gov), albeit with adverse effects. Given the promising effects observed herein with low OPN5 concentration (0.1 μM), a lower dose of OPN5 can be considered to minimize adverse effects while retaining immunomodulatory activity. OPN5 is a modestly BD1-selective BET inhibitor, and research with other BET inhibitors indicates that BD1-selective inhibitors may have increased efficacy in immunomodulation, especially at lower doses [45,52]. Our study underscores the translational potential of epigenetic-based therapeutics like BET inhibitors to target MDSCs, but further promotion of other BET inhibitors, such as BD2-specific inhibitors that retain efficacy but exhibit lower toxicity, may be better suited for clinical use. Additionally, various studies have utilized BET inhibitors in combination with targeted small-molecule inhibitors, immune checkpoint inhibitors, and other epigenetic-based therapies, indicating a future for BET inhibitors as useful component of combinatorial approaches to cancer therapy [45]. For example, our group has demonstrated that OPN5 exhibits synergistic cytotoxicity with venetoclax—a front-line BCL2-targeting therapy for CLL—supporting its use as a combination therapy [53]. This could open possibilities to utilize BET inhibitors as a multi-faceted therapeutic that can also target MDSC-mediated immune suppression in cancer. While our findings support the rationale for employing BET inhibitors in reshaping the TME, a comprehensive evaluation of clinical dosing strategies and feasibility warrants further investigation. Nonetheless, our results lay a strong foundation for future translational studies aimed at optimizing BET inhibitor dosing regimens in clinical settings, especially in combination with immunotherapies or targeted agents.

Finally, due to the diminished immune-suppression capacity of MDSCs isolated from OPN5-treated mice, we sought to further characterize TME T-cell populations in these mice. Overall, OPN5 treatment did not influence percentages of splenic CD8+ or CD4+ T-cells; however, splenic T-cells from OPN5-treated mice displayed phenotypic changes. We found that T-cells from OPN5-treated mice exhibited decreased exhaustion phenotypes. Extending our analysis further, we evaluated populations of terminally exhausted, terminally differentiated, and stem-like T-cell phenotypes. We discovered OPN5 treatment decreased terminally exhausted (PD-1hi/TIM3hi) T-cells and terminally differentiated (PD-1+/TIM3+/Ly108−) CD8+ T-cells populations, while increasing the proportion of stem-like (PD-1+/TIM3−/Ly108+) splenic CD8+ T-cells. These findings are in concordance with previous studies from our group that indicate OPN5 treatment can revive T-cell functionality [24]; however, this study contributes in another important way, as we additionally show that MDSCs from OPN5 treatment alleviate immune suppression towards T-cells. Through influencing tumor CLL B-cells, accessory MDSCs, and the T-cells themselves, OPN5 contributes to the revival of CLL-associated T-cells, which can have important implications in tumor control. OPN5-mediated reversal of T-cell exhaustion in CLL supports the use of BET inhibitors as important immunomodulatory agents, especially in combination with immune-based therapies such as checkpoint blockade or CAR-T therapy.

Overall, we demonstrated that BRD4 is overexpressed in MDSCs, which may contribute to MDSC development in CLL. Additionally, we show that pharmacological BET inhibition with OPN5 reverses MDSC-mediated immune suppression in preclinical models of CLL, highlighting the translational potential of BET inhibition as an anti-cancer therapeutic simultaneously targeting MDSCs in CLL.

## Supplementary Material

Supplemental File

**Supplementary Materials:** The following supporting information can be downloaded at: https://www.mdpi.com/article/10.3390/hemato6020014/s1, Supplemental Methods Table S1: Antibodies for immunoblotting; Supplemental Methods Table S2: Antibodies for flow cytometry; Supplemental Methods Figure S1: T-cell proliferation and IFNγ production flow cytometry gating strategy; Supplemental Methods Figure S2: Myeloid cell analysis flow cytometry gating strategy; Supplemental Data Figure S1: Ex vivo treatment with OPN5 changes bone marrow-derived MDSC populations; Supplemental Data Figure S2: Effects of ex vivo treatment with OPN5 on WT-derived MDSCs; Supplemental Data Figure S3: Additional Eμ-TCL1 adoptive transfer study to validate the in vivo effects of OPN5 treatment on CLL-associated MDSCs.

## Data Availability

Data will be made available from the authors upon reasonable request.
