# Supplementary material for: BET Protein Inhibition Relieves MDSC-Mediated Immune Suppression in Chronic Lymphocytic Leukemia
Source: Hemato. Author manuscript; Available in PMC 2025 Jul 24. (PMC12288560; doi:10.3390/hemato6020014)
Supplement: Supplemental File [file NIHMS2088910-supplement-Supplemental_File.pdf]

Supplemental File

I. Supplemental Methods

Supplemental Methods Table S1. Antibodies for immunoblotting.

Target	Supplier	Catalog Number	Source/Isotype
β-Actin	Santa Cruz Biotech.	47778	Mouse IgG
BRD4	Cell Signaling Tech.	83375	Rabbit IgG
Lamin-B1	ThermoFisher Scientific	702972	Rabbit IgG
Anti-mouse IgG	Cell Signaling Tech.	7076	Horse
Anti-rabbit IgG	Cell Signaling Tech.	7074	Goat

Supplemental Methods Table S2. Antibodies for flow cytometry.

BL = BioLegend, BD = BD Biosciences, TF = ThermoFisher Scientific.

Mouse-specific antibodies					Experiment			
**Target**	**Fluoro-chrome**	**Clone**	**Host and Isotype**	**Supplier**	**T-cell function**	**Myeloid analysis**	**T-cell analysis**	**CLL/MDSC blood flow**
B220	PerCP	RA3-6B2	Rat IgG2a, κ	BL				X
B220	PE/Cy7	RA3-6B2	Rat IgG2a, κ	BL				X
CD3	BUV496	145-2C11	Armenian Hamster IgG1, κ	BD		X		
CD3	PE/Cy7	145-2C11	Armenian Hamster IgG	BL				X
CD4	PerCP/Cy5.5	RM4-5	Rat IgG2a, κ	BL	X		X	
CD5	FITC	53-7.3	Rat IgG2a, κ	BL		X		X
CD8a	FITC	53-6.7	Rat IgG2a, κ	BL	X		X	
CD11b	BV785	M1/70	Rat IgG2b, κ	BL	X	X		
CD11b	FITC	M1/70	Rat IgG2b, κ	BL				X
CD19	BV605	6D5	Rat IgG2a, κ	BL		X		
CD19	PE	6D5	Rat IgG2a, κ	BL				X
CD43	BUV737	S7	Rat IgG2a, κ	BD		X		
CD45	AF700	30-F11	Rat IgG2b, κ	BL		X		
CD45	APC	30-F11	Rat IgG2b, κ	BL				X
CD84	PE	mCD84.7	Armenian Hamster IgG	BL		X		
CD101	AF700	Moushi101	Rat IgG2a, κ	TF			X	
IFNγ	PE	XMG1.2	Rat IgG1, κ	BL	X			
Lag3	APC	C9B7W	Rat IgG1, κ	BL			X	
Ly6C	BV510	HK1.4	Rat IgG2c, κ	BL		X		
Ly6C	PerCP	HK1.4	Rat IgG2c, κ	BL				X
Ly6G	BV650	1A8	Rat IgG2a, κ	BL		X		
Ly6G	PE	1A8	Rat IgG2a, κ	BL				X
Ly108	BV711	13G3	Mouse IgG2a, κ	BD			X	
PD-1	BV421	29F.1A12	Rat IgG2a, κ	BL			X	
PD-L1	BV421	10F.9G2	Rat IgG2b, κ	BL		X		
TIM3	BUV395	5D12	Mouse IgG1, κ	BD			X	
Zombie NIR™ Fixable Viability Dye				BL	X	X	X	

Supplemental Methods Figure S1

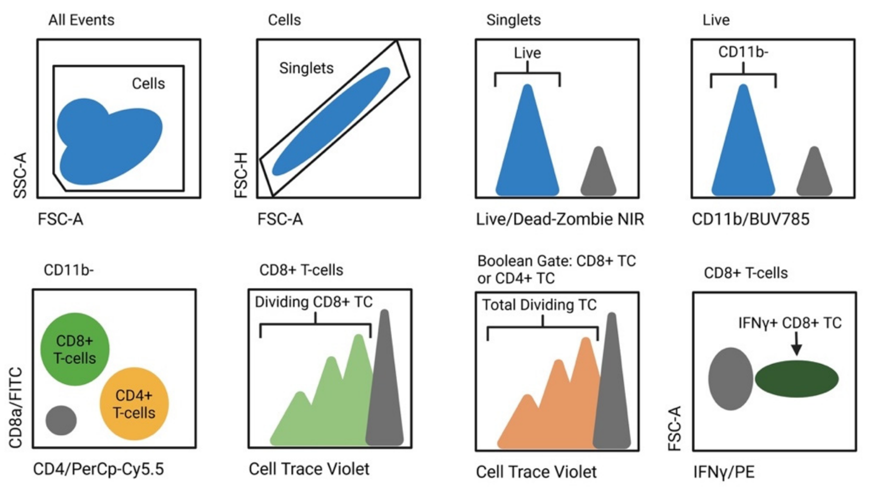

Supplemental Methods Figure S1. T-cell proliferation and IFNγ production flow cytometry gating strategy. TC = T-cells.

Supplemental Methods Figure S2

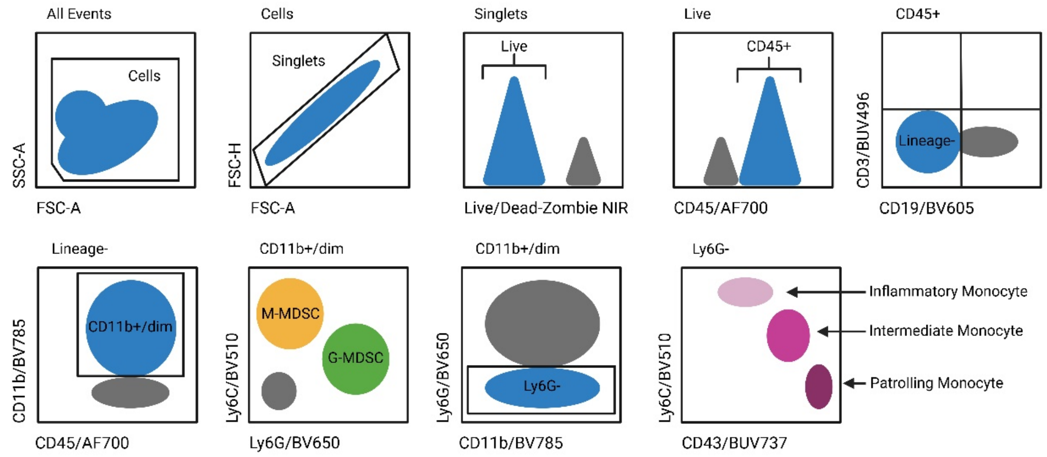

Supplemental Methods Figure S2. Myeloid cell analysis flow cytometry gating strategy. M-MDSC = monocytic myeloid-derived suppressor cell; G-MDSC = granulocytic myeloid-derived suppressor cell.

II. Supplemental Data Figures

Supplemental Data Figure S1

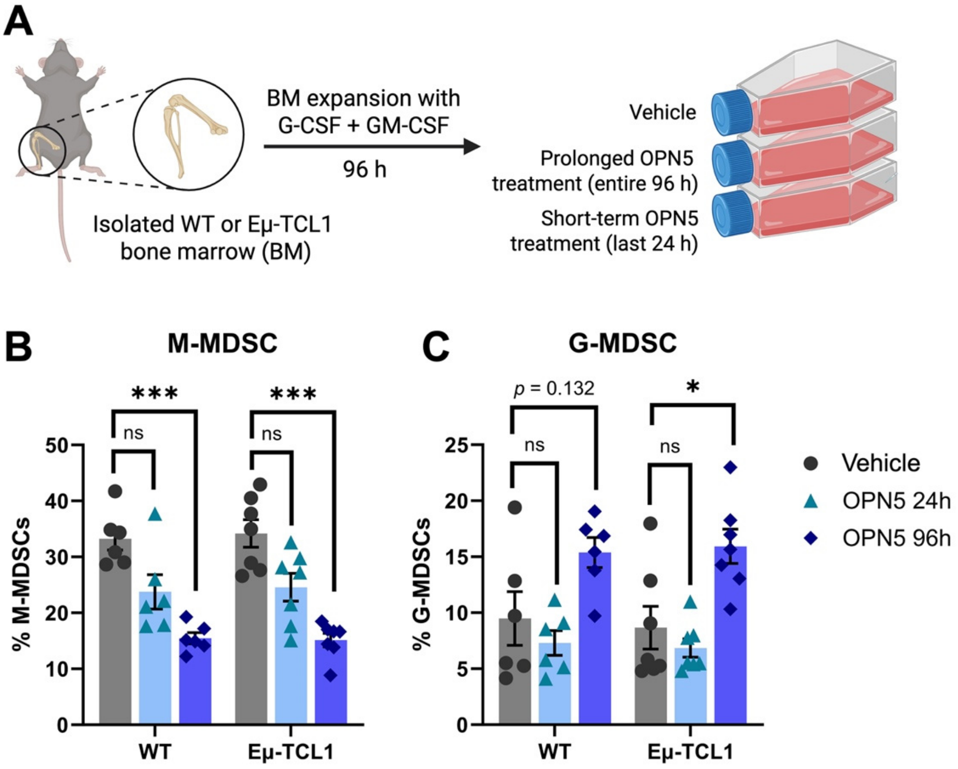

Supplemental Data Figure S1. Ex vivo treatment with OPN5 changes bone marrow-derived MDSC populations. **(A)** Workflow schematic demonstrating ex vivo expansion of bone marrow cells from C57BL/6J wild-type (WT) and Eμ-TCL1 mice. All conditions were cultured for 96 h in MDSC-supportive medium that contained 20 ng/mL G-CSF and 20 ng/mL GM-CSF in the presence or absence of OPN5. Vehicle treatment conditions received supportive medium without OPN addition. Prolonged OPN5 treatment conditions received 0.1 μM OPN5 for the entire 96 h expansion (OPN5 96 h). Short-term OPN5 treatment condition received 0.1 μM OPN5 for the last 24 h (OPN5 24 h). **(B–C)** M-MDSC populations are shown as percent of Ly6C+/Ly6G- cells out of CD45+/CD19-/CD3-/CD11b+ cells. G-MDSC populations are shown as percent of Ly6Clo/Ly6G+ cells out of CD45+/CD19-/CD3-/CD11b+ cells, as determined by flow cytometry, n=6–7 experimental replicates. Comparisons across treatment groups were analyzed with respect to vehicle control by one-way ANOVA. Data is presented as mean ± SEM; ns = not significant, * $p < 0.05$, ** $p < 0.01$, **** $p < 0.0001$. WT = wild-type, BM = bone marrow, OPN5 = OPN-51107.

Supplemental Data Figure S2

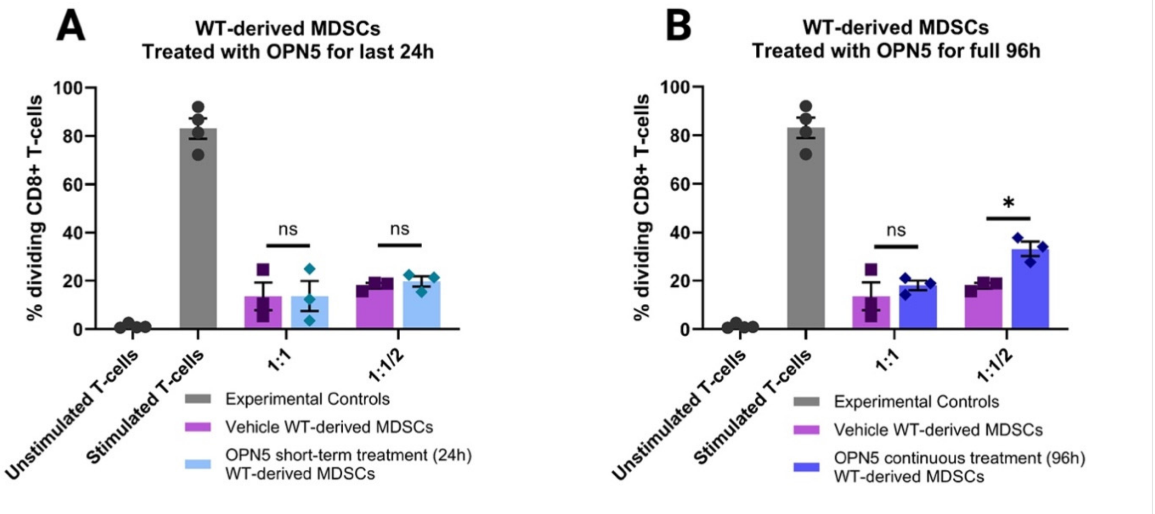

Supplemental Data Figure S2. Effects of ex vivo treatment with OPN5 on WT-derived MDSCs. **(A)** Bone marrow isolated from C57BL/6J wild-type (WT) mice (n=3) were expanded in MDSC-supportive medium. At end of 96 h expansion, MDSCs were isolated, and co-cultured with CTV-labeled healthy T-cells (isolated from WT mouse spleens) at 1:1 or 1:1/2 T-cell/MDSC ratio. To stimulate T-cell proliferation, 10 mg/mL plate-bound murine anti-CD3, 1 mg/mL murine anti-CD28, and 50 ng/mL murine IL-2 were added. The percentage of dividing T-cells is shown. Experimental controls (dark grey bars) include monocultures of unstimulated healthy T-cells and stimulated T-cells (anti-CD3/anti-CD28 with IL-2). WT-derived bone marrow expanded in MDSC supportive medium were subject to short-term BET inhibitor treatment (0.1 μM OPN5 for the last 24 h of expansion) and compared to vehicle control (n=3 experimental replicates). **(B)** WT-derived bone marrow expanded in MDSC supportive medium were subjected to prolonged BET inhibitor treatment (0.1 μM OPN5 for the full 96 h of expansion) and compared to vehicle control (n=3 experimental replicates). Data are presented as mean ± SEM. Unpaired Mann–Whitney U tests were used to determine the significance between treatment groups; ns = not significant, * $p < 0.05$. WT = wild-type, OPN5 = OPN-51107.

Supplemental Data Figure S3

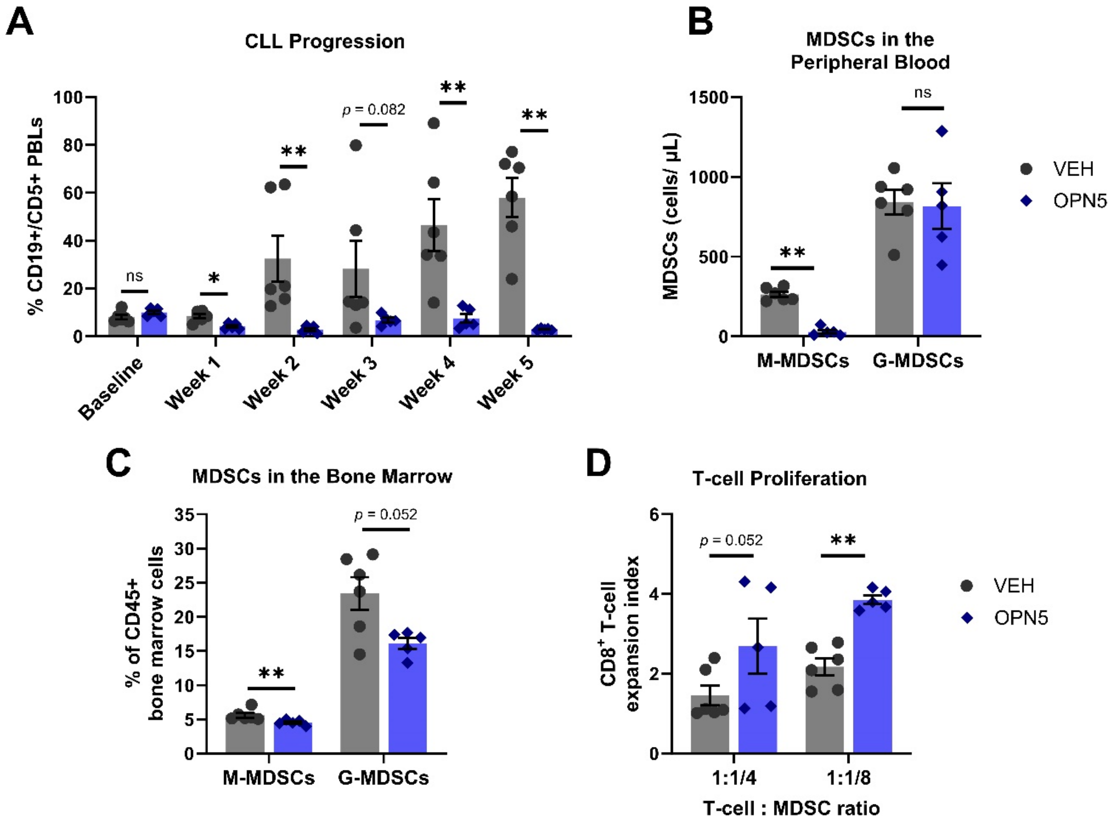

Supplemental Data Figure S3. Additional Eμ-TCL1 adoptive transfer study to validate the in vivo effects of OPN5 treatment on CLL-associated MDSCs**.** An aggressive adoptive transfer model was employed by engraftment of 1 x 10^7 spleen-derived lymphocytes from serially engrafted Eμ-TCL1 mice into recipient C57BL/6J wild-type (WT) mice. Once disease was evident in the peripheral blood [~10% CD45+/CD19+/CD5+ peripheral blood lymphocytes (PBLs)], mice were randomly assigned to receive daily treatment of 20 mg/kg OPN5 (n=5) or vehicle equivalent (n=6) daily, via oral gavage for a total of five weeks. Peripheral blood, bone marrow cells, and ex vivo co-cultures were evaluated by flow cytometry. **(A)** Percentage of CD45+/CD19+/CD5+ PBLs (CLL-like B-cells) are before treatment (Baseline) and across five weeks of treatment. **(B)** Absolute count of peripheral blood M-MDSCs (CD45+/CD19-/CD3-/CD11b+/Ly6C+/Ly6G- cells) and G-MDSCs (CD45+/CD19-/CD3-/CD11b+/Ly6Clo/Ly6G+ cells) at end of treatment. **(C)** Percentage of M-MDSCs and G-MDSCs in the bone marrow. **(D)** MDSCs (CD11b+/Gr1+) isolated from the bone marrow of treated mice and directly co-cultured with healthy T-cells to determine MDSC suppressive function. Healthy T-cells were isolated from a pool of splenocytes from 3-month-old WT mice and labeled with CTV for T-cell proliferation assays. Co-cultures were seeded at 1:1/4 and 1:1/8 T-cell/MDSC ratios and stimulated with 10 mg/mL plate-bound murine anti-CD3, 1 mg/mL murine anti-CD28, and 50 ng/mL murine IL-2. The expansion index, ratio of final cell counts to starting cell counts, of CD8+ T-cells is shown. Data are presented as mean ± SEM. Unpaired Mann–Whitney U tests were used to determine significant differences between VEH and OPN5 groups; ns = not significant, * $p < 0.05$, ** $p < 0.01$. VEH = vehicle equivalent, OPN5 = OPN-51107.
